# HELENA: HER2-Low as a prEdictive factor of response to Neoadjuvant chemotherapy in eArly breast cancer

**DOI:** 10.1186/s12885-022-10163-9

**Published:** 2022-10-20

**Authors:** François Cherifi, Angélique Da Silva, Alison Johnson, Cécile Blanc-Fournier, Olivia Abramovici, Antonin Broyelle, Christelle Levy, Djelila Allouache, Ioana Hrab, Carine Segura, Adeline Morel, Maud Villemin, Clémence Boscher, Coraline Dubot-Poitelon, Pauline Rottier, Justine Lequesne, George Emile

**Affiliations:** 1 Department of Clinical Research, CLCC Francois Baclesse, Caen, France; 2 Breast Cancer Unit, CLCC François Baclesse, Institut Normand du Sein, Caen, France; 3 Department of Pathology, CLCC Francois Baclesse, Caen, France; 4 Department of Pathology, CLCC Oscar Lambret, Lille, France; 5 Breast Cancer Unit, CLCC Oscar Lambret, Lille, France

**Keywords:** Antibody–drug conjugates, HER2-low, Breast cancer, Trastuzumab–deruxtecan, Neoadjuvant treatment, Lymphocytes, Neutrophils

## Abstract

**Background:**

HER2 expression has a prognostic and predictive impact in early-stage breast cancer (BC). HER2 positive BC (immunohistochemistry (IHC) score 3 + or 2 + with in situ hybridization (ISH) amplification) are treated with HER2 targeted therapies. The concept of HER2-low BC (IHC score 1 + or 2 + without ISH amplification) is drawing attention as anti-HER2 treatment has recently shown efficacy in this subgroup. We aimed to explore the response to neoadjuvant chemotherapy (NAC) in HER2-low early BC according to the HER2 score (1 + or 2 + without amplification).

**Methods:**

We conducted a retrospective study in two French comprehensive cancer centers. All patients with HER2-low BC treated with NAC from January 2014 to December 2020 were included. The primary objective was to analyze the pathological complete response (pCR) rate to NAC using the Sataloff or RCB system, according to the HER2 score. Secondary objectives were to assess disease free survival (DFS), overall survival (OS) and to explore the immune environment through the Neutrophil-to-Lymphocyte Ratio (NLR), according to HER2 expression. Univariate and multivariate analyses were performed.

**Results:**

We included 237 tumors for 229 patients. Of these, 160 (67.5%) tumors were HER2 1 + , 77 (32.5%) were HER2 2 + , and 152 (64.1%) were hormone receptor (HR) positive. The median age was 53.9 years. No differences in tumor characteristics were observed between HER2 1 + and HER2 2 + subgroups. pCR was achieved in 38 tumors (17%), without any difference between HER2 1 + and HER2 2 + subgroups (*p* = 0.77). DFS and OS were significantly different between HER2 1 + and HER2 2 + patients (HR = 0.41,CI95%[0.17;0.97] *p* = 0.037 and HR = 0.31,CI95%[0.09;1.02] *p* = 0.042, respectively). HER2 status was still associated with DFS and OS after adjustment for age, HR status and NLR, with better outcomes in favor of HER2 score 2 + (HR = 0.35 [0.15–0.84] and HR = 0.24 [0.07–0.81], respectively). NLR was not associated with worse DFS or OS.

**Conclusion:**

In HER2-low early BC, no differences in pCR were observed between HER2 1 + and HER2 2 + tumors, however patients with HER2 2 + tumors had a better DFS and OS than those with HER2 1 + . Further investigations are needed to describe the intrinsic differences in the spectrum of HER2-low BC.

## Introduction

Breast cancer (BC), the most commonly diagnosed cancer in women worldwide [1], is a heterogeneous disease, comprising distinct biological entities with different prognosis and oncogenic drivers. There are four primary clinical subtypes of BC: luminal A-like, luminal B-like, human epidermal growth factor receptor 2 (HER2) positive, and triple negative BC (TNBC) [2]. Historically, HER2-positive BC had a worse prognosis [3]. The development of agents targeting HER2 has provided significant clinical benefits and altered its natural course [4, 5]. HER 2 overexpression has also been described in other solid tumors (biliary tract, gastric carcinoma, bladder) for which HER2 targeted therapies are a standard treatment and/or in development [6, 7].

According to the French expert pathologists’ group (GEFPICS) [8] and ASCO [9] guidelines, HER2 score is evaluated by immunohistochemistry (IHC) with score 0 the absence of membrane marking, score 1 + if the marking is between 0 and 2 + , score 2 + a weak to moderate complete membrane staining in > 10% of tumor cells, and score 3 + a complete intense membrane marking in > 10% of cancer cells. In case of intermediate expression (score 2 +), additional research of HER2 gene amplification is performed by in situ hybridization (ISH). A distinction is made between HER2-positive tumors (score 3 + or 2 + with positive ISH), HER2-negative tumors (score 0) and HER2-low tumors which have a low expression of HER2 (i.e., score 1 + or 2 + /ISH negative) [8, 10–13]. HER2 positivity, found in approximately 15% of BC [14], is predictive of response to HER2 targeted treatments [15]. Until recently, HER2-low tumors were less well-defined and no specific treatment existed for this subgroup. HER2 scores 1 + and 0 are frequently grouped together under the denomination of “HER2 negative” [16], resulting in a likely underestimation of HER2 score 1 + and important variations in the reported proportions of HER2 score 0 ranging from 18 to 80% [8, 16]. With this limitation in mind, the proportion of HER2-low tumors is estimated to be between 45 and 55% of all BC. HER2-low BC are classified as either luminal if hormone receptors (HR) are positive or TN if HR are negative [13]. Several authors reported that in early BC, HER2 score 2 + [17–20] was associated with a poorer prognosis in comparison with HER2 score 0 or 1 + [18, 21]. The expression of HER2 is more a continuum than an on/off marker, but until now various studies carried out in neoadjuvant or adjuvant settings have not shown any benefit to adding an anti-HER2 treatment in HER2-low BC [13, 15, 22]. With the arrival of new antibody–drug conjugates (ADC) such as trastuzumab-deruxtecan or trastuzumab-duocarmazine, more treatment opportunities may be available [23]. In a phase Ib trial, trastuzumab-deruxtecan was effective in HER2-low advanced BC [24] and the FDA recently granted breakthrough therapy designation to trastuzumab deruxtecan for the treatment of HER2-low metastatic BC based on the Destiny-Breast04 trial results [25].

Furthermore, novel ADCs interact with the immune system [26],which plays a crucial role in cancer development. Inflammation could be reflected by pretreatment peripheral leukocyte counts and ratios such as the neutrophil-to-lymphocyte ratio (NLR). This biomarker appears to be an independent prognostic factor in BC patients treated with neoadjuvant chemotherapy (NAC), especially for HR positive BC [27, 28]. To date, no data are available for HER2-low BC.

In early BC, the effectiveness of NAC is evaluated by pathological complete response (pCR) rates. pCR has prognostic value and is one of the main treatment objectives [29]. Few data concerning pCR rates in patients with HER2-low early BC are available.

The primary objective of our study was to explore pCR rates in HER2-low early BC patients treated with NAC, according to HER2 status (score 1 + versus score 2 + , ISH negative). Secondary objectives were to compare disease free survival (DFS) and overall survival (OS) between these two subgroups and to explore NLR’s prognostic value in HER2-low BC patients.

## Material and methods

### Study design and population

We conducted a retrospective bicentric study which enrolled all HER2-low early BC patients treated with NAC at the Comprehensive Cancer Centers François Baclesse (Caen, France) and Oscar Lambret (Lille, France). Patients had to meet the following inclusion criteria: age ≥ 18 years, with histologically proven invasive HER2-low BC, and who received NAC followed by surgery. HER2 expression was classified according to ASCO guidelines [12]. HER2-low status was defined as HER2 IHC score of 1 + or 2 + without amplification by FISH/ISH testing. We excluded patients with HER2 score 0 and HER2 positive BC (i.e., HER2 score 3 + or score 2 + and FISH/ISH-amplified), those who received neoadjuvant endocrine therapy, those who did not receive the complete planned chemotherapy regimen and those who did not undergo surgery. HR were assessed by IHC on pretreatment biopsy. Tumors were defined as HR positive if estrogen receptor (ER) and/or progesterone receptor (PR) nuclear staining was positive in ≥ 10% of tumor cells.

### Data collection and outcomes

We collected the following clinicopathological characteristics at baseline: age, ECOG-PS at diagnosis, gender, menopausal status, blood counts, histological subtypes, TNM and histoprognostic factors (grade, HR status, HER2 status, Ki67 and vascular embolism) determined by biopsy. After surgery, assessment of pCR (by Sataloff [30] and/or RCB classification [31]), TNM, and histoprognostic factors (grade, HR status, HER2 status, Ki67, vascular embolism) determined on the resected specimen were collected, as well as data concerning treatments (surgery, chemotherapy regimen and safety) and follow-up (recurrence of cancer or death). The primary objective of this study was to evaluate pCR rates according to HER2-low status. Secondary objectives were to assess disease free survival (DFS), i.e., the time between the diagnosis to the date of any clinical or radiological relapse, overall survival (OS), i.e., the time between the diagnosis to death from any cause (or last follow-up), according to HER2-low status (score 1 + versus score 2 +), to explore the prognostic value of the neutrophil to lymphocyte ratio (NLR) defined as the absolute neutrophil count divided by the absolute lymphocyte count pre-chemotherapy, and to analyze the safety.

### Statistical analysis

Statistical tests and confidence intervals were calculated with an alpha risk level of 5%. Descriptive analyzes were provided for the qualitative variables by frequencies and percentages and for quantitative variables by the median and extreme values.

The characteristics of HER2 score 1 + and HER2 score 2 + patients were compared by χ2 test (or Fisher's exact test, in case of observed values per category < 5) for the qualitative variables, and by the Student's t-test for the quantitative variables (or Wilcoxon non-parametric test if data were not normally distributed). DFS and OS were estimated by the Kaplan–Meier method, and comparison of survival between different patient populations was performed by the log-rank test. Multivariable analysis for DFS and OS was performed using Cox's proportional hazards regression model, including HER2 status and factors significantly associated with survival at a significance level of 0.10. NLR was dichotomized by an optimal cut-off value, computed to predict progression or death during the follow-up with the highest product of sensitivity and specificity, through receiver operating characteristics (ROC) curve analysis. Analyses were conducted using R software, version 4.0.2 [32].

### Statement of ethics

This project was approved by the institutional review board of the cancer center François Baclesse, Caen, France. Informed consent was obtained from all individual participants included in the study. It was conducted in compliance with the French Research Standard MR-004 “Research not involving Human participants”. It is registered in the French Health Data Hub under the reference N° F20210125175632.

## Results

### Patients’ characteristics

**Fig. 1:**
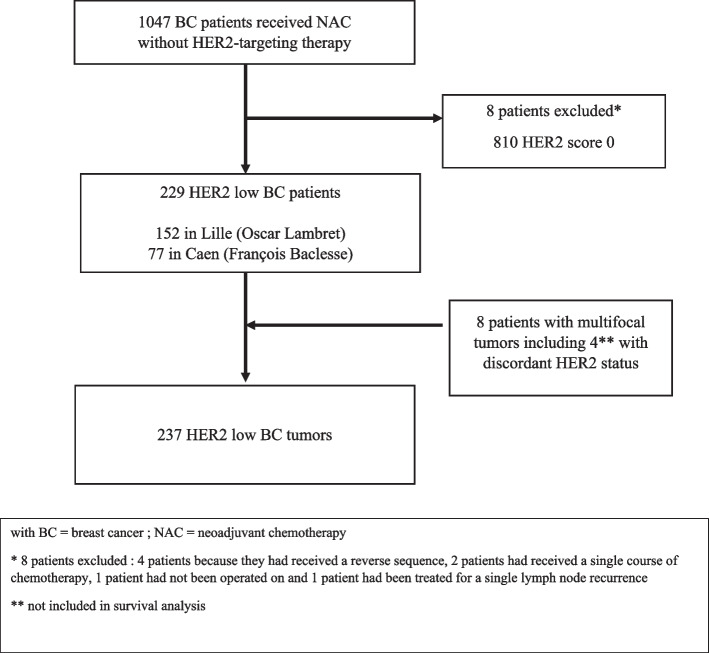
Flow diagram

From January 2014 to December 2020, 1047 patients received NAC in both centers. Of these, 229 had HER2-low BC and were enrolled in this study. Due to the existence of multifocal or bilateral tumors, patients had a total of 237 analyzed tumors. Among these, 160 (67.5%) tumors were HER2 score 1 + and 77 (32.5%) were HER2 score 2 + (Fig. 1). The baseline characteristics of patients are summarized in Table 1. The median age was 51 (range, 24–83). Most tumors (90%) were classified as invasive carcinoma of No Special Type (NST). Median tumor size was 40 mm (range, 28 – 51 mm) before NAC and half of patients had nodal involvement (53.5%). Most tumors were grade 3 (51.9%) and 56.1% had a Ki67 superior to 20%. Sixty-four percent of tumors were HR positive. Patients were slightly older, with a median age of 56 (range, 47–66) versus 49.5 (range, 41–60) *p* = 0.0016, and therefore more often menopausal (*p* = 0.05) in the Caen center. Tumor size was also smaller in patients from this center, with a median size of 40 mm (range, 30–60) versus 35 mm (range, 28–45) *p* = 0.015. Characteristics were similar between HER2 score 1 + and HER2 score 2 + subgroups (see Table 2).

**Table 1:** Patient characteristics

Variable	n	%	N available
Sex			229
Female	228	99.6	
Male	1	0.4	
Age, years [range]	51 [24–83]		229
Menopause			228
Yes	118	51.8	
No	110	48.2	
Histology			237^a^
NST	214	90.3	
Lobular	17	7.2	
Other	6	2.5	
HR			237^a^
positive	152	64.1	
negative	85	35.9	
ER			237^a^
< 10%	88	37.1	
≥ 10%	149	62.9	
PR			237^a^
< 10%	134	56.5	
≥ 10%	103	43.5	
HER2			237^a^
1 +	160	67.5	
2 +	77	32.5	
Baseline NLR			226
> 1.96	104	46.0	
< 1.96	122	53.9	
Stage			237^a^
IA	8	3.4	
IB	80	33.8	
IIA	84	35.4	
IIB	29	12.2	
IIIA	33	13.9	
IIIB	3	1.3	
Chemotherapy regimen			229
12 paclitaxel	3	1.3	
3 (F)EC—3 docetaxel	141	61.6	
3 (F)EC—9–12 paclitaxel	48	21.0	
4 (F)EC—4 docetaxel	5	2.2	
4 (F)EC—9–12 paclitaxel	15	6.6	
4 carboplatinum-paclitaxel—4EC	1	0.4	
4 carboplatinum-paclitaxel—4AC	1	0.4	
4–6 docetaxel—cyclophosphamide	2	0.9	
6 docetaxel—epirubicine	11	4.8	
6 docetaxel	1	0.4	
6 carboplatinum—paclitaxel	1	0.4	
Surgery			229
Partial mastectomy + SLN biopsy	43	18.8	
Partial mastectomy + ALND	72	31.4	
Total mastectomy + SLN biopsy	91	39.7	
Total mastectomy + ALND	17	7.4	
Total mastectomy without ALND	6	2.6	
Response to neoadjuvant chemotherapy			
Sataloff score			213
Complete response = TANA / TANB	37	17.4	
No pCR	176	82.6	
RCB score			106
Complete response = 0	15	14.2	
No pCR	91	85.8	

*With NST* No Special Type, *ER* Estrogen receptor, *PR* Progesterone receptor, *HER2* Human epidermal growth factor receptor 2, *NLR* Neutrophil-to-Lymphocyte Ratio, *(F)EC* (fluorouracil) – epirubicin – cyclophosphamide, *AC* Adriamycin – cyclophosphamide, *RCB* Residual breast Cancer Burden, *ALND* Axillary lymph node dissection, *pCR* Pathological complete response, *SLN* Sentinel lymph node

^a^ Corresponding to the number of tumors

**Table 2:** Tumor characteristics according to HER2 status

	HER2 1 +			HER2 2 +			*p*-value
	*N* = 160			*N* = 77			
Variable	n	%	N	n	%	N	
SBR grade			160			77	0.48
I	5	3.1		1	1.3		
II	69	43.1		39	50.6		
III	86	53.8		37	48.1		
ER IHC expression			160			77	0.58
< 10%	57	35.6		31	40.3		
≥ 10%	103	64.4		46	59.7		
PR IHC expression			160			77	0.79
< 10%	89	55.6		45	58.4		
≥ 10%	71	44.4		32	41.6		
Ki67 IHC expression			121			55	0.44
≤ 20%	27	22.3		16	29.0		
> 20%	94	77.7		39	71.0		
Lymphovascular embolism			112			54	0.95
Yes	29	25.9		13	24.1		
No	83	74.1		41	75.9		

*With HER2* Human epidermal growth factor receptor 2, *SBR* Scarff-Bloom and Richardson, *ER* Estrogen receptor, *PR* Progesterone receptor, *IHC* Immunohistochemistry

The optimal NLR cut-off to predict progression or death was 1.96 (Area Under the Curve 0.58, sensitivity of 59.5% and specificity of 57.2%); baseline NLR was below 1.96 in 122 patients (54%, *N* = 226).

### Response to neoadjuvant chemotherapy

The majority of patients (*n* = 219; 96%) received a sequential combination of anthracyclines and taxanes as NAC; 114 (49.7%) patients underwent total mastectomy and 115 (50.3%) underwent conservative breast surgery. Sentinel lymph node dissection was performed in 134 (58.5%) patients and axillary lymph node dissection in 89 (38.8%) patients (Table 1). There were no differences in the treatment approaches between the two centers. Assessment of the pathological response to NAC was available according to the Sataloff classification or RCB score for 224 tumors. pCR was achieved in 38 tumors (17%), of which 24 (16.1%) were HER2 score 1 + and 14 (18.7%) were HER2 score 2 + (*p* = 0.77, see Table 3).

**Table 3:** Response to neoadjuvant chemotherapy and survival according to HER2 status

	HER2 1 +			HER2 2 +			*p*-value
	*N* = 160			*N* = 77			
Variable	n	%	N	n	%	N	
Sataloff score			143			70	0.61
TANA / TANB	23	16.1		14	20		
No pCR	120	83.9		56	80		
RCB score			69			37	1
0	10	14.5		5	13.5		
No pCR	59	85.5		32	86.5		
RCB or Sataloff score			149			75	0.77
pCR	24	16.1		14	18.7		
No pCR	125	83.9		61	81.3		

*HER2* Human epidermal growth factor receptor 2, *RCB* Residual breast Cancer Burden, *pCR* Pathological complete response

### Survival analysis

The median follow-up time was 30 months (range 6.2- 84.3). A total of 42 patients experienced disease recurrence or death during follow-up, with a 3-year DFS rate of 80.4% [95% Confidence Interval (CI95%) 74.5–86.9]. Twenty-seven (12%) patients died during follow-up, with a 3-year OS rate of 87.1% [81.6–92.8]. Four patients who presented HER2 score 1 + and 2 + tumors were excluded from the survival analysis.

### Univariable Cox analysis

**Fig. 2:**
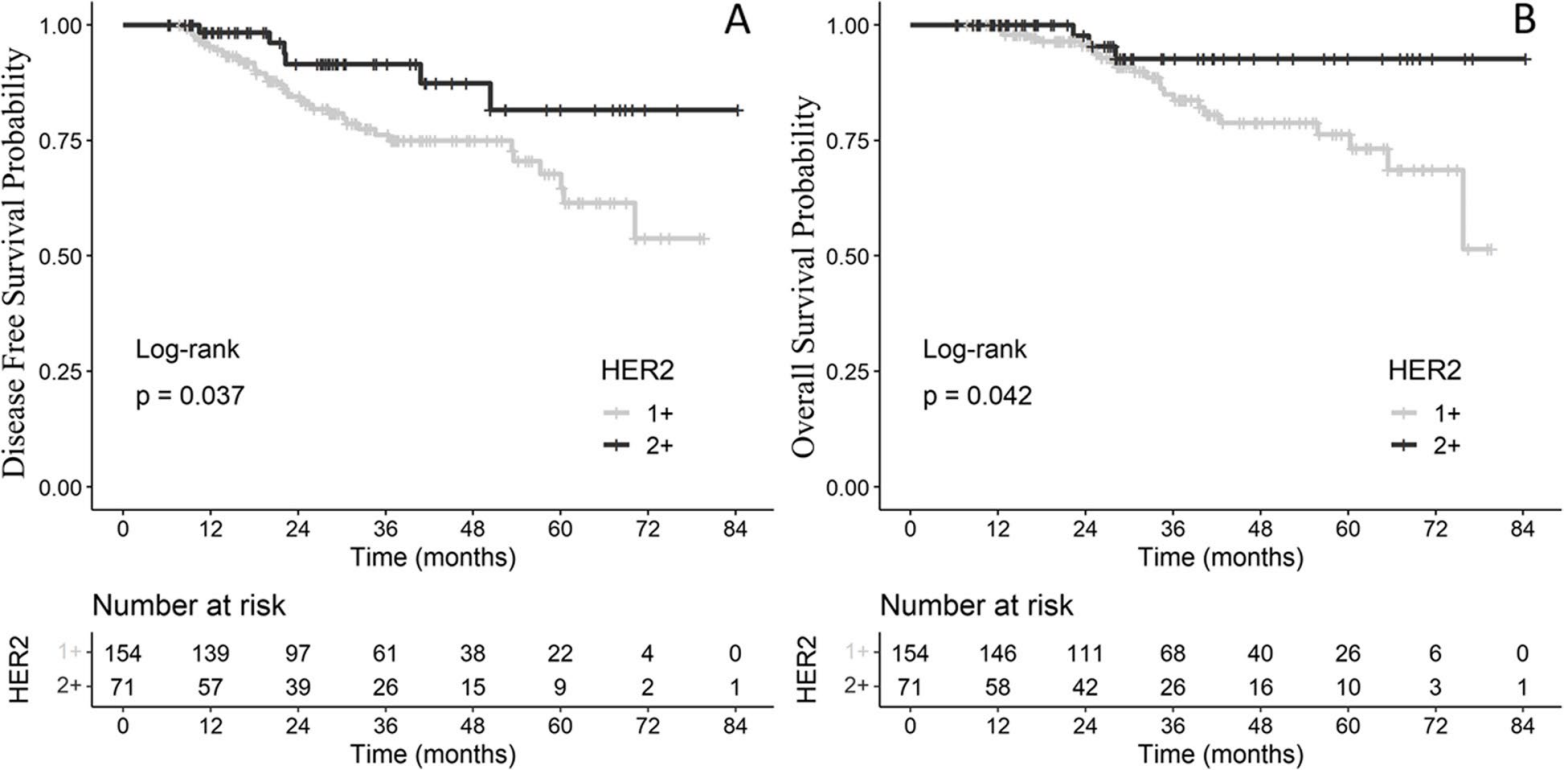
DFS (**A**) and OS (**B**) according to HER2 low status

DFS was significantly different according to HER2 subgroup (log-rank *p* = 0.037), with HR = 0.41, CI95% = [0.17;0.97] in favor of HER2 score 2 + (Fig. 2A and Table 4). No statistically significant difference in DFS was observed between patients with high NLR (≥ 1.96) and those with low NLR (< 1.96) (log-rank *p* = 0.096). Among the other tested factors, age and HR status were significantly associated with DFS (Table 4).

**Table 4:** Univariable and multivariable Cox analysis of progression free survival and overall survival

	PFS				OS			
	Univariable		Multivariable		Univariable		Multivariable	
	HR [95%CI]	Cox p	HR [95%CI]	Cox p	HR [95%CI]	Cox p	HR [95%CI]	Cox p
Age	1.03 [1.01–0.06]	**0,009**	1.03 [1.01–1.06]	**0,012**	1.05 [1.02–1.08]	**0,003**	1.09 [1.03–1.15]	**0,003**
Menopause	1.65 [0.89–3.05]	**0,11**			2.22 [1.01–4.86]	**0,047**	0.35 [0.08–1.50]	0,16
Histology (Lobular/other)	0.48 [0.12–1.99]	0,31			0.42 [0.06–3.10]	0,39		
HR positive	0.52 [0.28–0.95]	**0,035**	0.58 [0.31–1.07]	0,079	0.47 [0.22–1]	**0,05**	0.50 [0.22–1.10]	0,085
ECOG PS 1	0.69 [0.21–2.24]	0,54			1.12 (0.34–3.74]	0,85		
Lymph node involvement	1.28 [0.69–2.36]	0,44			1.66 [0.74–3.72]	0,22		
NLR < 1.96	0.59 [0.32–1.10]	**0,099**	0.56 [0.30–1.05]	0,072	0.54 [0.25–1.19]	**0,13**	0.56 [0.20–1.04]	0,062
HER 2 IHC 2 +	0.41 [0.17–0.97]	**0,043**	0.35 [0.15–0.84]	**0,019**	0.31 [0.09–1.02]	0,055	0.24 [0.07–0.81]	**0,022**
Baseline lymphopenia	1.24 [0.61–2.53]	0,55			1.43 (0.60–3.40]	0,42		

*HER2* Human Epidermal growth factor Receptor 2, *HR* Hormone Receptor, *NLR* Neutrophil-to-Lymphocyte Ratio

OS significantly differed according to HER2 subgroup (log-rank *p* = 0.042), with HR = 0.31, CI95% = [0.09;1.02] in favor of HER2 score 2 + (Fig. 2B and Table 4). NLR (dichotomized by a cut-off of 1.96) was not significantly associated with OS (log-rank *p* = 0.13). Age, menopausal status and HR positivity were significantly associated with OS (Table 4).

### Multivariable Cox analysis

In our multivariable model, HER2 status was still associated with DFS after adjustment for age, HR status and NLR, with better outcomes for patients with HER2 score 2 + BC (HR = 0.35 [0.15–0.84]). Although not associated above the significance level of 10% in univariable analysis, NLR was kept in the OS multivariable model as an adjustment factor. Especially, HER2 status was still associated with OS, independently of age, menopause, HR status and NLR (HR = 0.24 [0.07–0.81]). Note that NLR < 1.96 was not significantly associated with better DFS and OS outcomes in multivariable models ( Cox *p* = 0.072 and *p* = 0.062, respectively).

### Safety

Sixty-four (27.9%) patients experienced an adverse event (AE); most (58.1%) were grade 1–2. The most frequently reported AE were skin/mucosal toxicity (*N* = 11, 17.2%) and hematologic toxicity (*N* = 10, 15.6%). Dose reductions were necessary for 43 (18.8%) patients.

## Discussion

HER2-low early stage BC appears to be a distinct biological entity. In this study, we further highlighted this fact by demonstrating that, inside the subgroup of HER 2-low BC, HER2 score 2 + is an independent positive predictive marker for tumor recurrence and survival after neo-adjuvant therapy. To date, few data are available on the intrinsic differences inside the HER2-low subgroup in this setting.

In our two cancer centers, among the entire population who received neo-adjuvant therapy during the study period, 22.6% of tumors were classified as HER2-low with 15.3% HER2 score 1 + and 7.4% HER2 score 2 + . In comparison, from January 2014 to December 2021, the HER2 French database, a platform collecting data on different tumors from all French comprehensive cancer centers, registered 19.3% of HER2 score 1 + and 24.0% of HER2 score 2 + tumors before NAC [33].

To our knowledge, this is the first study to compare the intrinsic differences between HER2- scores of 1 + and 2 + in early BC treated with NAC. There is a high risk of confounding HER2 IHC score 0 and score 1 + . In the literature, the proportion of HER2 IHC score 0 varies from 18 to 80% of BC [13, 16]. In a retrospective setting, there is a significant risk of bias by falsely classifying HER2 score 0 as HER2 score 1 + or vice versa. The consequence could be the dilution of a possible difference between HER2 score 0 and HER2-low. In a large pooled analysis of 2310 patients in NAC clinical trials, Denkert et al. [34] identified HER2-low tumors in 47.5% of patients, which is more than the 20% in our study and is possibly due to the centralized review of the HER2 score often used in clinical trials.

We selected patients referred for neoadjuvant chemotherapy, which is often recommended, in the context of HER2 negative BC, for TNBC [35]. HER2-low tumors are more frequently HR positive [13, 21] which we confirmed in our cohort with 65% of HR positive tumors. This relatively high proportion of HR positive BC may explain our low pCR rate of 17%, which is lower than expected for TNBC or HER2 positive tumors [35]. pCR may be a good surrogate endpoint for DFS and OS and can be used for early drug approval but precaution is still required [29, 36]. The latest St Gallen international consensus guidelines do not recommend pCR as the main objective for neoadjuvant treatment studies [37]. De Moura Leite et al. did not find any differences in pCR or survival (DFS and OS) between HER2-low and HER2 score 0 tumors in a retrospective setting [38]. However, Denkert et al. found in their pooled analysis that HER2-low tumors had a significantly lower pCR rate (10%) than HER2 score 0 tumors, particularly in the HR positive subgroup. They also showed better 3-year DFS and OS for HER2-low than for HER2 score 0 tumors [34]. It may be interesting to determine the prognosis of the specific subgroup of patients with HER2 score 2 + in this population.

In an ancillary study of the I-SPY 2 TRIAL, quantitative measurement of HER2 protein was positively associated with response to ado-trastuzumab emtansine (T-DM1) and pertuzumab in patients already identified as HER2 positive [39]. This reinforces the hypothesis that HER2 scoring should be interpreted as a continuum: the more it is expressed, the better the predictive value.

Other studies have also suggested better outcomes in HER2-low versus HER2 score 0 tumors [34]. In our study, we found better survival rates for HER2 score 2 + BC patients than for HER2 score 1 + . If this survival difference is not due to the initial tumor response to NAC, we can hypothesize that the difference may be due to a more intrinsic aggressiveness of HER2 score 1 + BC, with a stronger tendency to micro metastasize or develop a resistance to treatment, however this requires specific confirmatory studies. A crosstalk exists between the ER and HER2 signaling pathways, which can lead to treatment resistance [40, 41]. One of the strengths of our study is that our results remain positive, independently of the HR status.

NLR is a simple biomarker of growing interest in BC. High NLR could help identify patients with a poor response to NAC [28]. High NLR (> 2.25) has been shown to be an independent prognostic factor for worse DFS and OS in HR positive/HER2-negative BC patients receiving NAC [27]. In another study, high pretreatment NLR (≥ 2.5) was also significantly associated with shorter DFS in all patients, and shorter OS in Luminal A BC [42]. However, these two studies did not specifically explore the HER2-low subgroup. In our study, a high NLR (> 1.96) was not associated with worse prognosis, probably due to the lack of power. There was no difference in NLR levels between patients with HER2 score 1 + and score 2 + .

Our study has several limitations, including its retrospective nature and relatively small number of events despite a bi-centric design. The initial design of our study was to find a difference in pCR and not in survival, however there was no obvious reason to fear selection bias reversing our conclusion. Our HER2 status was obtained by biopsy, which remains a partial reflection of potential tumor heterogeneity. In HER2 positive patients, HER2 heterogeneity is a predictor of lower pCR rates after targeted neoadjuvant treatment [43] and highly heterogeneous tumors are associated with significantly shorter DFS [44]. Another limitation is the observer variability in the interpretation of HER2 status, due to the absence of a central pathological review and patient inclusion across a large time period with different guidelines for HER2 testing and interpretation. As mentioned previously, the distinction between scores 0 and 1 + , which was until recently without any therapeutic impact, results in a large interindividual variability [8]. This may have led to an underestimation of our HER2-low population and explain the small proportion we report compared to national data. This can be mitigated by the fact that the majority of analyses took place in two major French comprehensive cancer centers. There is excellent agreement (98—99%) in the evaluation of positive HER2 status between biopsy and surgical specimen, but this is not necessarily the case for HER2-low BC.

New ADC targeting HER2, such as trastuzumab deruxtecan or trastuzumab duocarmazine, have shown pre-clinical efficacy in HER2-low breast cancer cells. These effects may be due to a higher drug-antibody ratio, driving a greater amount of cytotoxic payload to the targeted cells and a bystander effect [23, 24, 45, 46]. Recently, the FDA granted Breakthrough Therapy Designation for trastuzumab deruxtecan in HER2-low metastatic BC following the positive results of the DESTINY-Breast04 trial [25]. Many studies are in progress and focus on HER2-low tumors (NCT04556773, NCT04494425, NCT04742153 and NCT05165225) [45]. The aim of these studies is to extend the survival benefits observed with anti-HER2 agents in HER2-positive disease to a greater proportion of patients with HER2-low BC [45, 46].

In the coming years these novel treatments will likely be the standard of care for patients with HER2-low BC. Future studies will certainly evaluate different treatment sequences for HER2-low BC patients to determine the optimal use of anti-HER2 agents and other targeted therapies such as Sacituzumab-govitecan, which has shown efficacy in HER2-low BC and is approved in pre-treated metastatic TNBC [47]. The emergence of the HER2-low group with a specific treatment requires a more reproducible or novel way of assessing HER2 expression, as a quantitative measurement, ranging from HER2 0 to HER2 3 + , with new subcategories such as HER2 ultra low currently being studied [46]. This could also increase the need for post-operative assessment of HER2 status [8, 39]. Our discovery of survival differences based on a subtle change in the HER2 status underlines the need to better understand the mechanisms of this variable expression of HER2 [40, 41, 48].

## Conclusion

In early stage HER2-low BC, HER2 score 2 + /ISH non-amplified is an independent predictive marker for better DFS and OS after neoadjuvant chemotherapy compared to HER 2 score 1 + . HER2 score 1 + and HER2 score 2 + are distinct entities emphasizing that the HER 2 score is not a binary entity but a continuum. Although these results need to be confirmed in a larger prospective study, they encourage us to consider personalized treatments for patients with HER2-low BC.

## Data Availability

The datasets used and analysed during the current study are available from the corresponding author on reasonable request.

## Abbreviations

ADC: Antibody–drug conjugate
BC: Breast cancer
CI: Confidence interval
DFS: Disease Free Survival
ISH: In Situ hybridization
PFS: Progression free survival
HER2: Human epidermal growth factor receptor 2
HR: Hormone receptor
NAC: NeoAdjuvant Chemotherapy
NLR: Neutrophil to Lymphocyte
NST: No Special Type
OS: Overall survival
pCR: Pathologic complete response
RCB: Residual breast Cancer Burden
TNBC: Triple negative breast cancer
